# Finger joint laxity, number of previous pregnancies and pregnancy induced back pain in a cohort study

**DOI:** 10.1186/1471-2393-14-61

**Published:** 2014-02-06

**Authors:** Anne Lindgren, Per Kristiansson

**Affiliations:** 1 Department of Public Health and Caring Sciences, Family Medicine and Preventive Medicine Unit, Uppsala University, SE-751 22 Uppsala, Sweden; 2 Department of Physiotherapy, Sundsvall Hospital, SE-851 85 Sundsvall, Sweden

**Keywords:** Finger joint laxity, Back pain, Pregnancy, Postpartum

## Abstract

**Background:**

General joint hypermobility is estimated to affect about 10% of the population and is a prerequisite of heritable connective tissue disorders where fragile connective tissue is a prominent feature. Pregnancy induced back pain is common whereas about 10% of women still have disabling pain several years after childbirth. The pathogenesis of the pain condition is uncertain, although several risk factors are suggested including general joint hypermobility. In the present study, the possible association of peripheral joint mobility in early pregnancy on the incidence of back pain with onset during pregnancy and persisting after childbirth was explored.

**Methods:**

A cohort of 200 pregnant women recruited from antenatal health care clinics was assessed by questionnaire and clinical examination, including measurement of passive abduction of the left fourth finger, throughout pregnancy and at 13 weeks postpartum. Comparisons were made between women with and without back pain. Statistical tests used were χ^2^-test, t-test, Spearman correlation and multiple logistic regression.

**Results:**

In the cohort, the mean passive abduction angle of the left fourth finger increased from 40.1° in early pregnancy to 41.8° at the postpartum appointment. At the postpartum appointment, women in the back pain group had a significantly larger mean passive abduction angle of the left fourth finger of 4.4°, twice as many previous pregnancies and deliveries, and more than twice as frequent back pain in previous pregnancy, as compared with women with no persistent back pain. A similar pattern was displayed in late pregnancy. In a multiple regression analysis, the passive abduction angle of the left fourth finger in early pregnancy and the number of previous pregnancies were positively, significantly and independently associated to the incidence of back pain in late pregnancy and postpartum.

**Conclusions:**

Finger joint laxity as a reflection of constitutional weakness of connective tissue and number of previous pregnancies were associated with the development of back pain induced in pregnancy and persisting after childbirth. These factors may provide a foundation for development of targeted prevention strategies, but this have to be confirmed in future research including measurement of general joint laxity.

## Background

General joint hypermobility is estimated to affect about 10% of the population and is a prerequisite of heritable connective tissue disorders where fragile connective tissue is a prominent feature [1-4]. The cause of the higher prevalence of general joint hypermobility among women is uncertain however suggested to be related to sex-specific adaptations of the connective tissue [5]. Throughout pregnancy, with dramatic hormonal changes, an increased mobility of peripheral joints with partial reversion after delivery has been reported [6-8].

One in three women in the world experience back pain induced during pregnancy with the number of previous deliveries as a strong determinant [9]. Other known determinants of pregnancy induced back pain include early menarche, hormone contraceptive use before first pregnancy, physically demanding work and emotional distress [10-12]. In addition, hormonal and reproductive factors have been associated with risk of chronic low back pain [13].

In most women, the back pain disappears soon after delivery [14,15]. However, in about 8% of women disabling pregnancy-induced back pain continues several years after delivery, even up until old age [15,16], that entails severe changes of everyday life activities [17]. Characteristics of women with slow regression of back pain include early onset, longer periods of pain and higher intensity of pain during pregnancy, and work dissatisfaction, disbelief in improvement and emotional distress, and heavy work situation regardless of pregnancy [14,18-21].

The connective tissue of the low back and pelvis are fundamental to transmitting body forces between the axial skeleton and the lower extremities. Results from several studies indicate that pregnancy affects the connective tissue in general and in the pelvic region particularly [22,23]. Thus, hormonal influence on particularly fragile connective tissue could be an important factor in the development of pregnancy-induced back pain persisting after childbirth. In a previous study self-reported joint hypermobility was a determinant of back pain persistent after childbirth [24].

In the present study, using previously studied data, we hypothesized that peripheral joint laxity, as a proxy of general joint hypermobility, measured in early pregnancy was associated with pregnancy-induced back pain in late pregnancy and three months postpartum.

## Methods

### Study population

All pregnant women living in two districts of the city of Sundsvall, Sweden, were identified through check-ups at the antenatal care units in the metropolitan area, at the offices of practicing gynecologists and at the outpatient clinic at the local hospital. All Caucasian and Swedish speaking women attending during early pregnancy in 1991 were sampled for this study. Two hundred and twenty-seven pregnant women fulfilled the sampling criteria, of whom 222 attended the antenatal care units serving the two districts. These 222 women were invited to participate in the study. Twenty-two declined participation, which left 200 (88.1%) women as the final study population. All women were apparently healthy, and none had ongoing medication. During the follow-up period, 10 women left the study because of spontaneous abortion, two women declined further participation, and one woman moved from the area.

### Methods

The methods for this back pain study have been described in detail previously [14]. Briefly, data were collected at three appointments during pregnancy, on average at 11 (range 6 to 19), 24 (range 21 to 27) and 36 (range 34 to 38) completed gestational weeks, and at a fourth appointment 13 (range 4 to 29) weeks postpartum. Early pregnancy was defined as gestational week 11. At each appointment, the women completed a questionnaire and underwent a general clinical examination including measurement of the passive abduction angle of the left fourth finger. For all women, the duration of pregnancy was confirmed by ultrasonography in estimated gestational week 19 and was registered as completed weeks of gestation.

### Medical history

The questionnaire included an instrument for measuring ongoing pain, its location, intensity, and consequences in terms of disability. In addition, there were questions about previous obstetric history, previous back pain problems and current smoking habits. The questionnaire was completed in privacy with no time limit and was checked for completeness.

Women who reported pain were instructed to indicate the location of the pain on a pain drawing. More than one location could be indicated. The back locations were coded as cervical spine, thoracic spine, lumbar spine, and sacral spine. In the present study, women who reported any of these four back pain locations were pooled into a group labeled “back pain” and women who reported neither of these back pain locations were pooled into a group labeled “no back pain”, at each study visit. The women were also asked to estimate the date of onset of pain for each pain location. Only one onset time was registered at each appointment. If several back pain locations were indicated, the time of onset of the lowest back pain location was registered.

The intensity of pain at the moment and the worst pain during the past week, was described on two visual analogue scales (VAS): 0 mm indicating no pain and 100 mm indicating intolerable pain [25].

Twelve disability ratings measuring the ability to perform activities of daily living were recorded on VAS scales, where 0 mm indicated no restriction on the activity and 100 mm indicated inability to do the activity. The mean score of the 12 ratings was used as the disability rating index (DRI) [26].

### Clinical examination

**Figure 1:**
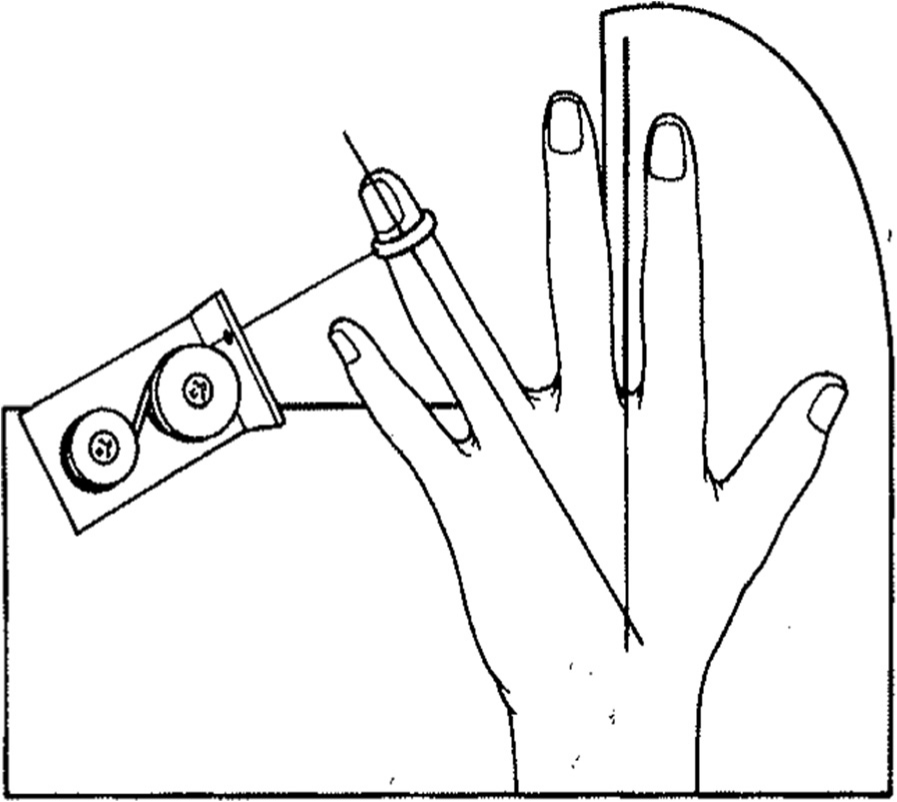
**Device used to assess the passive abduction angle of the left fourth finger.** The angle between the immobilized second finger and the fourth finger with a force of 1.7 Newton applied to the medial side of the distal phalanx was recorded. Reproduced with permission from HC Ostgaard [28].

At each of the four appointments, the passive abduction angle of the left fourth finger was measured with a well defined and constant abduction force of 1.7 Newton applied to the medial side of the distal phalanx, with the forearm and hand in horizontal position and the second finger immobilized (Figure 1). The angle between the fourth and second finger was measured using a protractor to the nearest degree by the same physician throughout the study. The same method has been used before assessing abduction angle of the left fourth finger in early pregnancy [27] and a significant correlation between abduction angle of left fourth finger and Beighton score, was reported (r = 0.55, p < 0,001) [28]. The reliability of the abduction angle measurements was calculated by the intra-individual coefficient of variance. The coefficients of variance between the first and second measurement was 0.077, between the second and third 0.070 and between the third and fourth 0.071. The coefficient of variance of all four angle measurements across the nine months study period was 0.085.

At study inclusion weight and height were measured. Height was measured barefoot with a wall-mounted tape measure to the nearest centimeter. Weight was measured with indoor clothing on a balance lever scale with digital presentation of kilograms to one decimal point. Body mass index was calculated as weight (kg)/height^2^ (m^2^).

No systematic pain treatment was given, but women with severe pain were offered sick listing or physiotherapy, which included sacroiliac belt and pool training.

### Ethics and consent

Permission for this study was obtained from the Research and Ethics Committee of the University of Umeå and all women gave their informed consent. The procedures followed were in accordance with the ethical standards of the responsible committee on human experimentation and with the Helsinki Declaration of 1975, as revised in 1983.

### Statistical analyses

Descriptive data were given as frequencies, percentages, means and standard deviations (SD). Spearman correlation was used to test association of inter-individual abduction angles at different time intervals. Differences between the dependent intra-individual abduction angles at different time intervals were tested with paired t-test. Comparison between groups regarding continuous data was performed using t-test and comparisons of categorical data with χ^2^. Only two-tailed tests were used and a p-value <0.05 was considered statistically significant. No account was taken of multiple comparisons.

**Figure 2:**
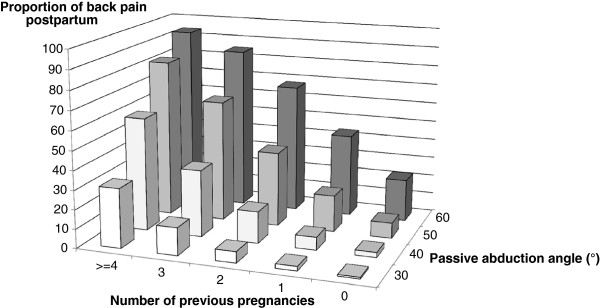
**Finger joint laxity, number of pregnancies and back pain persisting after childbirth.** The association of finger joint laxity and number of previous pregnancies with pregnancy induced back pain incidence persisting three months after delivery. The logistic regression model was used to compute expected mean incidence estimates of back pain.

To control for possible confounding, multiple logistic regression analysis was used and P-values and confidence intervals (CI) were given. Time since last delivery was excluded from the regression analysis since it excessively reduced the total number of women included in the analysis. The set of possible determinants in early pregnancy were: passive abduction angle of the left fourth finger, age, weight, height, number of previous pregnancies, current smoking habits and reported previous back pain problems. The categorization of the nominal and ordinal factors used in the model were 0, 1, 2, 3 or ≥4 previous pregnancies, no current smoking (0), smoking <10 cigarettes/day (1) and smoking ≥10 cigarettes/day (2) and yes/no for previous back pain problems. For the analyses of the regression surface in Figure 2, the logistic regression model was used to compute expected mean incidence estimates of back pain postpartum, based on the passive abduction angle of the left fourth finger in early pregnancy and the number of previous pregnancies. To estimate the ability of the passive abduction angle of the left fourth finger measurement to predict the risk of developing pregnancy-induced back pain, the receiver operating characteristic curve and logistic regression were used. In a post-hoc power calculation with a β-risk of 0.80, a significance level of 0.05 and a standard deviation of 6.5° indicated that a study population of 200 women would detect a difference in the abduction angle of the left fourth finger of 2.6°. The results were obtained using the Statistical Analysis System, version 9.3, SAS Institute Inc, Cary, NC, USA.

## Results

Characteristics in early pregnancy of the women included in the study are shown in Table 1.

**Table 1:** Characteristics in early pregnancy of the women included in the study

**Characteristic**	**n**	**Mean (S.D.) or proportion (%)**
Age (yr)	195	27.8 (4.6)
Weight (kg)	188	63.7 (8.7)
Height (m)	187	1.66 (0.06)
Body mass index (kg/m^2^)	187	23.0 (2.9)
No cigarette smoking (%)	151/195	77
Pregnant for the first time (%)	79/194	41
Nulliparous (%)	116/194	60
Number of previous pregnancies	194	0.9 (0.9)
Number of previous deliveries	194	0.5 (0.7)
Time since previous delivery (yr)	78	3.3 (0.4)
Previous back pain, not during pregnancy (%)	113/195	58
Back pain in previous pregnancy (%)	46/115	40

The mean passive abduction angle of the left fourth finger increased from 40.1° in early pregnancy to 41.8° at the postpartum appointment (1.7°) (p < 0.0001), Table 2. In addition, a wide dispersion of the mean passive abduction angle of the left fourth finger was displayed, while the individual measurements at the four appointments were highly inter-correlated (0.68 < r < 0.76, p < 0.0001) (the latter data not shown).

**Table 2:** Abduction angle of the left fourth finger (°) throughout pregnancy and post-partum

**Time**	**n**	**Range**	**Mean (S.D.)**	**p value**
G.w. 11	194	24 to 62	40.1 (7.0)	
G.w. 24	187	28 to 64	41.0 (6.8)	
G.w. 36	173	24 to 59	40.6 (6.9)	
13 w pp	167	26 to 58	41.8 (7.0)	
Change from g.w. 11 to 24	186		0.9 (5.5)	0.02
Change from g.w. 24 to 36	172		-0.4 (4.9)	0.18
Change from g.w. 36 to 13 w pp	155		0.9 (5.2)	0.03
Change from g.w. 11 to 13 w pp	167		1.7 (5.2)	<0.0001

p-values refer to the intra-individual mean difference between the time points.

Gw = gestational week, w pp = weeks post partum.

Legend: Mean and range of abduction angle of the left fourth finger (°) among all women measured at the different time points during pregnancy and post partum. Also, the change of abduction angle of the left fourth finger between the different time points during pregnancy and postpartum are shown among those women with values at the particular time points. The statistical differences were tested with paired t-test.

The point prevalence rate of women reporting back pain with onset during the present pregnancy increased from 19% in gestational week 11, to 47% and 49% in gestational weeks 24 and 36, respectively, with a clear decline to 9% (16 women) at the postpartum appointment. At this particular time point, 14 of these 16 women reported pain located in the sacral back region and 2 reported pain in the lumbar region. The mean pain intensity at the postpartum appointment among those 16 women, 38.1 mm (SD 31.3), was significantly higher (p < 0.0001) than the mean pain intensity of 12.4 mm (SD 23.4) reported by the 154 women without back pain at this time. Another significant difference (p = 0.04) was shown for the disability rating index, 13.3 (SD 17.5) and 3.4 (SD 9.0), respectively, for the pain and no-pain groups postpartum.

Factors measured in early pregnancy for women with and without back pain with onset during their recent pregnancy and persistent three months after delivery are shown in Table 3. Women in the back pain group had a significantly larger passive abduction angle of the left fourth finger of 4.4°, twice as many previous pregnancies and deliveries and more than twice as great a frequency of back pain in previous pregnancy than women with no back pain. No significant differences between the groups were shown regarding the change of the abduction angle from early pregnancy to postpartum, age, weight, height, cigarette smoking, and previous back pain, irrespective of pregnancy. Similar differences between women with and without back pain with onset during the present pregnancy were shown at the appointment in late pregnancy, except for no significant difference of back pain in previous pregnancy (data not shown).

**Table 3:** Factors measured in early pregnancy by women with and without persistent back pain post partum

	**Back pain group**		**No back pain group**		
	**n**	**Mean (SD) or n (%)**	**n**	**Mean (S.D.) or n (%)**	**p value**
AA in early pregnancy (°)	16	44.1 (6.3)	154	39.7 (7.0)	0.019
AA pp – AA early pregnancy (°)	16	1.0 (7.1)	150	1.8 (5.0)	0.69
Age (yr)	16	29.1 (5.5)	155	27.7 (4.4)	0.25
Weight (kg)	16	67.0 (6.9)	154	63.4 (8.9)	0.12
Height (m)	16	1.66.1 (0.05)	153	1.66 (0.06)	0.86
Body mass index (kg/m^2^)	16	24.4 (2.8)	153	22.9 (2.9)	0.06
Cigarette smoking (%)	16	12 (2/16)	155	23 (35/155)	0.18
No. of previous pregnancies	16	1.6 (1.1)	154	0.8 (0.8)	0.0006
No. of previous deliveries	16	1.0 (0.8)	154	0.46 (0.6)	0.002
Time since previous delivery (yr)	11	3.5 (3.7)	60	3.3 (3.7)	0.88
Previous back pain, not in pregnancy (%)	16	44 (7/16)	155	57 (88/155)	0.13
Back pain in previous pregnancy (%)	13	77 (10/13)	89	34 (30/89)	0.003

AA = passive abduction angle of left fourth finger, pp = post partum.

Legend: Factors measured in early pregnancy by women reporting back pain induced in pregnancy and persisting 3 months post partum and those without such pain. Numbers of previous pregnancies and deliveries, abduction angle in early pregnancy and reported back pain in previous pregnancy were factors that significantly differed between the groups.

The associations between factors measured in early pregnancy and reported back pain with onset during present pregnancy and persistent postpartum are displayed in Table 4. In the univariate logistic regression analyses the abduction angle of the left fourth finger and number of previous pregnancies were significantly positively associated with reported back pain. Similar effects were shown between the different factors and back pain with onset during present pregnancy reported in gestational week 36.

**Table 4:** Association between factors in early pregnancy and back pain induced in pregnancy persistent post partum

	**Crude**			**Adjusted**		
**Characteristic**	**OR**	**CI**	**p value**	**OR**	**CI**	**p value**
AA in early pregnancy (°)	1.09	1.01-1.17	0.02	1.15	1.05-1.26	0.003
Age (yr)	1.07	0.96-1.19	0.25	1.02	0.89-1.17	0.81
Body mass index (kg/m^2^)	1.15	0.99-1.33	0.06	1.18	0.99.1.41	0.07
Cigarette smoking (%)	0.48	0.12-1.92	0.30	0.22	0.04-1.28	0.09
No. of previous pregnancies	2.34	1.37-3.98	<0.0001	3.24	1.57-6.68	0.002
Previous back pain (%)	0.59	0.21-1.67	0.32	0.68	0.21-2.20	0.52

AA = Passive abduction angle of the left fourth finger. OR = odds ratio. CI = 95% confidence interval.

Legend: Association between factors measured in early pregnancy on reported back pain with onset during present pregnancy and persisting 3 months post partum in several univariate and one multiple logistic regression analysis (n = 167). The abduction angle of the left fourth finger in early pregnancy and the number of previous pregnancies were significantly and independently associated to the incidence of back pain.

To find determinants for reported back pain with onset during the present pregnancy and persisting postpartum a multiple logistic regression analysis was performed, with all factors measured in early pregnancy included as independent variables, Table 4. The abduction angle of the left fourth finger in early pregnancy and the number of previous pregnancies were both positively, significantly and independently associated with the incidence of back pain. The concordance of the model was 83%.

Mean back pain incidence estimates based on the passive abduction angle of the left fourth finger in early pregnancy and the number of previous pregnancies was computed using the logistic regression technique and illustrated in Figure 2. Women with the greatest passive abduction angle of the left fourth finger and the highest number of previous pregnancies showed the highest back pain incidence, and vice versa. When the most extreme observed combinations of passive abduction angle of the left fourth finger and number of previous pregnancies were used, the highest incidence of back pain was estimated to 94% and the lowest to 0.8%.

The ability of the passive abduction angle of the left fourth finger measurement in early pregnancy to predict the risk of development of back pain with onset during pregnancy persisting 3 months postpartum was calculated using the receiver operating characteristic curve. At the optimal cut-off angle of approximately 40°, the sensitivity and specificity were 0.42 and 0.63, respectively. In a logistic regression analysis with back pain as the dependent variable and the finger angle as the independent variable the area under the curve was 0.66 and the odds of an event of back pain postpartum increased by a factor of 2.1 (C.I. 1.1-4.4) for each 10° increase of the angle (p = 0.03).

## Discussion

The number of previous pregnancies and the fourth finger joint laxity in early pregnancy, but not the change of joint laxity throughout pregnancy or previous back pain, were positively and independently associated to the incidence of pregnancy-induced back pain reported in late pregnancy and three months after delivery. The association of finger joint laxity might be as a reflection of general joint laxity in pregnancy and postpartum, which could be used in future preventive care. On the individual level, the ability of the finger laxity measured in early pregnancy to predict back pain incidence was moderate.

The association between peripheral joint laxity measured in early pregnancy and back pain induced in pregnancy and persisting after delivery, has not been presented before to the best of our knowledge. However, in an epidemiological study the women reporting a diagnosis of perceived joint hypermobility had a 1.5 times greater risk of having low back pain persistent 6 months after childbirth, which is in accordance with our results [24]. In contrast to our findings, a similar previous study displayed an inverse association between joint mobility in early pregnancy and reported back pain in pregnancy, although restricted to women pregnant for the first time [27]. In that study, the same method of finger joint mobility measurement was used but without taking into account whether the back pain started before or during the present pregnancy, which might explain the divergent results. The association between pregnancy induced back pain and number of previous pregnancies and deliveries has been established previously [9].

The association of finger joint laxity on development of back pain in pregnancy might be as a reflection of general joint laxity in pregnancy and postpartum, which in turn reflects a constitutional weakness of connective tissue [2,29]. The concept that the degree of joint laxity of any one individual is generalized throughout the body and that the majority of the stiffness of the metacarpophalangeal joints of the hand is a result of the capsule ligament complex and not the muscle-tendon units supports this view [30], as well as reported high correlations between finger joint laxity and general joint mobility measures [27,31,32]. In parallel, a constitutional connective tissue weakness in the pelvic region is suggested as a cause in women presenting with genitourinary prolapse and urinary incontinence [33,34].

Strengths of the present study were the high participation rate, the low drop-out rate, inclusion of only Caucasian women, assessment of only the left hand and one person measured the finger joint laxity at all time periods. In addition, possible confounding factors were controlled for in the multiple regression analyses.

There were several limitations of this study. Information of the validity of the used angle measurement device was limited although reassuring [28]. Stability in repeated angle measurements of the device was ascertained by intra-individual coefficient of variance between 0.07 and 0.08 in the present study. In previous studies of finger joint mobility two papers have presented reliability as coefficients of variance. In these studies lower coefficients of variance (1.5% and 3.6%) were presented with tests repeated within the same day as compared to several months in the present study [35,36]. Also, the wide dispersion of the time since delivery might have distorted the data, since recovery might be expected 29 weeks after delivery but not necessarily after 4 weeks. In addition, the number of women with persistent back pain after delivery was small and there was no information of socio-economic data. All these limitations probably reduced the sensitivity of the study to show only the strongest associations, and suggest that the chance of a false positive in detecting the shown associations is small.

Our finding of an association between peripheral joint laxity and pregnancy-induced back pain needs to be confirmed in future research, including measurement of general joint laxity measurement with high reliability, such as Beighton score [37].

## Conclusions

Finger joint laxity as a reflection of constitutional weakness of connective tissue and number of previous pregnancies were associated with the development of back pain induced in pregnancy and persisting after childbirth. These factors may provide a foundation for the development of targeted prevention strategies for women in early pregnancy at risk of developing disabling back pain after childbirth.

## Competing interests

The authors declare that they have no competing interests.

## Authors’ contributions

AL: conceived the study and contributed to analyses and interpretation of data and drafted the manuscript. PK: conceived the study, participated in its design, coordination and acquisition of data, contributed to analyses and interpretation, and drafted the manuscript. Both authors read and approved the final manuscript.

## Pre-publication history

The pre-publication history for this paper can be accessed here:

http://www.biomedcentral.com/1471-2393/14/61/prepub
